# Factors associated with dislocation after total hip arthroplasties performed for nontraumatic osteonecrosis of the femoral head: a multicenter cohort study of 5,983 hips

**DOI:** 10.2340/17453674.2025.43473

**Published:** 2025-04-17

**Authors:** Seneki KOBAYASHI, Nobuhiko SUGANO, Wataru ANDO, Wakaba FUKUSHIMA, Kyoko KONDO, Takashi SAKAI

**Affiliations:** 1 The Investigation Committee on Osteonecrosis of the Femoral Head under the Ministry of Health, Labour and Welfare, Tokyo; 2 Department of Orthopaedic Surgery, Suwa Red Cross Hospital, Suwa; 3 Department of Orthopaedic Medical Engineering, Osaka University Graduate School of Medicine, Suita; 4 Department of Orthopaedic Surgery, Kansai Rosai Hospital, Amagasaki; 5 Department of Public Health, Osaka Metropolitan University Graduate School of Medicine, Osaka; 6 Research Support Platform, Osaka Metropolitan University Graduate School of Medicine, Osaka; 7 Department of Orthopedic Surgery, Yamaguchi University Graduate School of Medicine, Ube, Japan

## Abstract

**Background and purpose:**

Nontraumatic osteonecrosis of the femoral head (ONFH) patients are at a higher dislocation risk after primary total hip arthroplasties (THAs) than osteoarthrosis patients. It has not been clear how large prosthetic heads should be to reduce dislocation. A nationwide multicenter follow-up cohort study of THAs performed for ONFH aimed to evaluate risk factors associated with dislocation and whether larger head size could reduce the dislocation risk.

**Methods:**

A multivariable logistic regression model analyzed factors associated with dislocation in 5,983 THAs performed for ONFH between 1996 and 2022 with a median of 7.1 (0.5–27)-year follow-up. Patient age at surgery was 52 years and BMI was 22.9, as medians. A posterior approach was employed in 59%. The head diameter was 22 mm in 4%, 26 mm in 15%, 28 mm in 24%, 32 mm in 36%, and ≥ 36 mm in 21%.

**Results:**

288 THAs (4.8%) dislocated. Younger (1^st^ quartile, ≤ 41 years) patient age (odds ratio [OR] 1.45 CI [95% confidence interval] 1.02–2.07 vs. 2^nd^ quartile), higher BMI (OR 1.05, CI 1.02–1.08 per 1), posterior approach (OR 3.33, CI 1.96–5.56 vs. anterior or anterolateral approach, OR 2.27 CI 1.59–3.23 vs. lateral approach), and smaller heads were identified as risk factors. However, ≥ 36-mm heads were not different from 32-mm heads (OR 1.06 CI 0.69–1.63).

**Conclusion:**

Risk factors associated with dislocation were younger patient age, higher BMI, posterior approach, and smaller heads; however, 32-mm heads were large enough to reduce dislocation.

Dislocation is 1 of the 3 most common reasons for revision after primary total hip arthroplasties (THAs) [1-3]. Larger prosthetic heads have been used increasingly often in THAs, and 32 mm and 36 mm are the most commonly used head sizes, as reported in national registries [1-3]. A report using data from the Nordic Arthroplasty Register Association database compared revision risk among head sizes in metal-on-polyethylene THAs performed for primary osteoarthrosis (OA). Highly cross-linked polyethylene (HXLPE) liners were matched with 32- and 36-mm heads in 75% and 95%, respectively, and there was a greater risk of aseptic loosening with 36-mm heads than 32-mm [4]. In the Australian Registry, in primary THAs performed for OA, with HXLPE liners, 32-mm heads had a lower revision rate for any reason than < 32-mm heads and than > 32-mm heads [3]. Larger heads have been shown to reduce the dislocation risk [5,6]. However, it has not been clear how large they should be to reduce dislocation.

Patients with nontraumatic osteonecrosis of the femoral head (ONFH) who undergo THAs are generally younger, more likely to be of male gender, larger in physique [7], and at a higher risk of postoperative dislocation [8-10] compared with OA patients. Results of THAs performed mainly for OA are not necessarily applicable to those for ONFH. Therefore, THAs performed for OFNH should be monitored, which has been conducted by the present nationwide multicenter follow-up cohort study. Many studies analyzed risk factors of dislocation after THAs performed mainly for OA. However, to our knowledge, there has not been a factor analysis including head size as a variable performed in a large cohort of ONFH patients. We aimed to evaluate risk factors associated with dislocation and whether larger head size could reduce the dislocation risk in patients with ONFH following a primary THA.

## Methods

### Study design

This is a prospective cohort study. The Investigation Committee on ONFH under the auspices of the Ministry of Health, Labour and Welfare set up a nationwide multicenter follow-up cohort study of primary THAs performed for ONFH to systematically clarify patient features, operative variables, and outcomes of the arthroplasties including postoperative dislocation. We studied factors associated with dislocation after primary THAs performed for ONFH.

The study was reported according to the STROBE guidelines.

### Setting and data source

Hip surgeons at 31 institutions (listed in Acknowledgements) participated in the study, registering, performing hip arthroplasties on, following up the ONFH patients, and collecting the data. We studied primary THAs performed at these hospitals for ONFH or OA secondary to ONFH between January 1996 and December 2022.

### Study population

Diagnosis and staging of ONFH was made according to the criteria of the committee [11]. Each ONFH patient who underwent THA was registered and followed clinically and radiographically at each institution and the follow-up status was reported to the committee every year. Criteria eligible for the study were not having a THA with very poor survivorship and ≥ 0.5-year follow-up.

### Variables

Recorded demographic data were age, gender, height, weight, body mass index (BMI), ONFH-associated factors (systemic steroid use and excessive alcohol consumption), ONFH stage, and previous surgery in the index hip joint. Surgery-related data encompassed approach, acetabular and femoral components (categorized by surface finish and use of cement in fixation), material of the acetabular articulating surface, and material and diameter of the femoral head. Follow-up information included postoperative dislocation (the outcome of the study) and the need for reoperation.

### Statistics

Risk factors were analyzed for postoperative dislocation with a multivariable logistic regression model using SAS version 9.4 (SAS Institute Inc, Cary, NC, USA). The normality of distribution was examined with the Kolmogorov–Smirnov test. A chi-square test examined the relationship between the variables. Univariable analyses were first performed applying the model to each of the demographic and operative variables with a significance level of P < 0.1. Relationship between candidates with P < 0.1 was analyzed, and from those with a strong association, 1 of them was deleted. Any candidate that was difficult to associate with dislocation without previous reports was also excluded. The remaining candidates were then analyzed together using the model with a significance level of P < 0.05 (multivariable analysis). IBM SPSS statistics version 29 (IBM Corp, Armonk, NY, USA) was also used to perform statistics including a chi-square test, t-test, and one-way analysis of variance, with a significance level of P < 0.05. To determine the threshold of head diameter that minimized dislocation risk (the secondary objective), the receiver operating characteristic (ROC) curve was plotted.

### Ethics, data sharing plan, use of AI, funding, and potential conflicts of interest

Ethical approvals for this study were comprehensively obtained at 3 representative institutions: Shinshu University School of Medicine (January 8, 2008, No. 1043), Suwa Red Cross Hospital (November 27, 2014, No. 26-23 and March 26, 2019, No. 30-19), and Osaka University Graduate School of Medicine (January 29, 2021, No. 20461). This study was carried out in accordance with the World Medical Association Declaration of Helsinki. All participants included in the study were informed and agreed to participate in this study and to have their data published in a journal. The data that supports the findings for this study is available to other researchers from the corresponding author upon reasonable request. AI was not used in the writing of this manuscript. This study was supported by a research grant from the Health Labour Sciences Research Grant, the Ministry of Health, Labour and Welfare, Japan (23FC0201). Except for the funding, the authors received or will receive no financial or material support for the research, authorship, and/or publication of this article. Complete disclosure of interest forms according to ICMJE are available on the article page, doi: 10.2340/17453674.2025.43473

## Results

**Figure 1:**
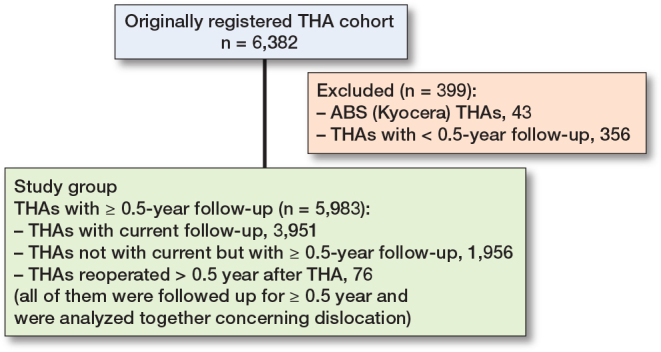
Flowchart of the study populations.

Dislocation risk was analyzed in 5,983 THAs (in 4,685 patients) with a median 7.1-year (0.5–27) follow-up (94% of the originally registered cohort of 6,382 THAs), excluding 43 ABS THAs with very poor survivorship and 356 THAs with < 0.5-year follow-up that was regarded as being not long enough to assess dislocation (Figure 1). The ABS THA (Kyocera, Kyoto, Japan) had a thin alumina liner supported by polyethylene in a socket. In our survey, it had a very low survival rate (62% at 10 years and 55% at 15 years). The study group of 5,983 THAs was composed of 3,951 THAs with current follow-up, 1,956 THAs not with current but with ≥ 0.5-year follow-up, and 76 THAs reoperated > 0.5 years after THA (due to recurrent dislocation in 20 hips, prosthetic joint infection 14, periprosthetic femoral fracture 11, and other reasons 31 [≤ 8 for each]). Reoperation was regarded as the end of follow-up. The 3,951, 1,956, and 76 THAs were followed up for ≥ 0.5 years and were analyzed together concerning dislocation.

Characteristics of the 5,983 THAs are listed in Table 1. With increasing head diameter, from 22–28 mm, to 32 mm, 36 mm, and 38–58 mm, the mean height (160.3, 161.8, 166.5, and 165.9 cm, respectively) became larger up to 36-mm (P < 0.001), but not between 36 mm and 38–58 mm (P = 0.8). The mean weight (59.0, 61.5, 66.0, and 65.2 kg, respectively) also became larger up to 36 mm (P < 0.001), but not between the latter 2 (P = 0.4). The 2 groups were associated with hospitals (P < 0.001). Acetabular articulating material was cobalt-chrome (i.e., metal-on-metal THA) in 1.1%, 0.5%, 2.9%, and 61.6%, respectively (P < 0.001). Neither resurfacing arthroplasties nor dual-mobility THAs were included in this study. During the follow-up, 288 THAs (4.8%) dislocated. Of these, 111 hips (39%) dislocated only once and 12 of them needed reoperation for dislocation in 4 and other reasons in 8. The remaining 177 hips (61%) had recurrent dislocations and 72 of the 177 (41%) needed reoperation for dislocation in 52 and other reasons in 20.

**Table 1:** Characteristics of the 5,983 THAs performed for nontraumatic osteonecrosis of the femoral head (ONFH)

Variable	Median [25–75 percentiles] (range) or n (%)
Patient age, years	52 [41–62] (15–93)
Height, cm	162 [155–169] (132–191)
Weight, kg	60 [52–69] (28–129)
Body mass index	22.9 [20.5–25.5] (11.6–42.4)
Male sex	3,220 (54)
ONFH-associated factor	
Systemic steroid use	3,609 (60)
Excessive alcohol consumption	1,607 (27)
Neither of them	636 (11)
Both of them	131 (2.2)
ONFH stage	
3 (collapse of the femoral head)	2,970 (50)
4 (osteoarthrosis)	2,854 (48)
2 (without collapse of the femoral head)	159 (2.7)
Previous hip surgery (joint-preserving surgery)	488 (8.2)
Surgical approach	
Posterior	3,499 (59)
Anterior or anterolateral	1,321 (22)
Lateral	1,163 (19)
Incision length	
Conventional	4,122 (69)
Minimum incision surgery	1,861 (31)
Uncemented fixation	
Acetabular component	5,844 (98)
Femoral component	5,119 (86)
Acetabular articulating material	
Highly **^a^** cross-linked polyethylene	3,665 (61)
Moderately **^b^** cross-linked polyethylene	1,264 (21)
Conventional polyethylene	559 (9.3)
Cobalt-chrome	266 (4.4)
Ceramic	229 (3.8)
Femoral head material	
Ceramic	3,798 (63)
Cobalt-chrome	1,732 (29)
Oxidized zirconium	340 (5.7)
Stainless steel	113 (1.9)
Head diameter, mm	
38–58 **^c^**	323 (5.4)
36	933 (16)
32	2,160 (36)
28	1,430 (24)
26	898 (15)
22	239 (4.0)

a approximately 10 Mrad.

b 5 to 7.5 Mrad.

c 38 mm in 8 hips, 40 mm in 67, 42 mm in 49, 44 mm in 44, 46 mm in 58, 48 mm in 40, 50 mm in 41, 52 mm in 12, 54 mm in 3, and 58 mm in 1.

### Risk factors for dislocation

The univariable analyses identified 8 of the variables listed in Table 1 at P < 0.1 (Table 2). Femoral component fixation, difficult to associate with dislocation, without previous reports (to our knowledge), and having a relation with 6 of the other 7 candidates, was not included in the next multivariable analyses. Femoral head material was related with 5 of the other 6 candidates. Its relationship with dislocation could not be found in the literature, although a hard-on-hard bearing was reported regarding dislocation, which was analyzed with acetabular articulating material. Therefore, femoral head material was not included either. As the remaining 6 variables had been reported concerning dislocation, and without a strong relationship between them, they were examined together with the model.

**Table 2:** Univariable analysis of each variable applying the logistic regression model to the 5,983 THAs performed for ONFH

Variable	Odds ratio (CI)	P value
Patient age, years (ref.: 2nd quartile [42–51])		
1st quartile (≤ 41)	1.35 (0.96–1.90)	0.08
3rd quartile (52–62)	1.15 (0.81–1.63)	0.4
4th quartile (≥ 63)	1.15 (0.81–1.64)	0.4
Male sex (ref.: female)	0.93 (0.74–1.18)	0.6
Height, cm (ref.: increment of 1)	1.00 (0.98–1.01)	0.5
Weight, kg (ref.: increment of 1)	1.01 (1.00–1.01)	0.3
Body mass index (ref.: increment of 1)	1.05 (1.02–1.08)	0.003
ONFH-associated factor (ref.: neither of them)		
Systemic steroid use	1.25 (0.82–1.91)	0.3
Excessive alcohol consumption	1.16 (0.74–1.82)	0.5
Both of them	0.54 (0.16–1.80)	0.3
ONFH stage (ref.: 4 osteoarthrosis)		
2 or 3 (before osteoarthrosis)	0.96 (0.75–1.21)	0.7
Previous hip surgery (ref.: no)	1.63 (1.13–2.35)	0.009
Surgical approach, posterior		
(ref.: anterior or anterolateral)	5.00 (3.03–7.69)	< 0.0001
(ref.: lateral)	1.82 (1.30–2.50)	< 0.0001
Incision length (ref.: conventional)		
Minimum incision surgery	0.45 (0.33–0.61)	< 0.0001
Acetabular component fixation (ref.: cemented)		
Uncemented	0.73 (0.30–1.79)	0.5
Femoral component fixation (ref.: cemented)		
Uncemented	1.36 (1.00–1.85)	0.05
Acetabular articulating material ref.: non-polyethylene)		
Polyethylene **^a^**	1.36 (0.29–1.77)	0.1
Femoral head material (ref.: metal)		
Ceramic	1.46 (1.15–1.85)	0.002
Head diameter, mm (ref.: 32)		
≥ 36	0.92 (0.60–1.41)	0.7
28	2.35 (1.67–3.30)	< 0.0001
26	3.51 (2.47–4.98)	< 0.0001
22	6.00 (3.81–9.42)	< 0.0001

THAs = total hip arthroplasties; ONFH = nontraumatic osteonecrosis of the femoral head; CI = 95% confidence interval.

a Including highly or moderately cross-linked polyethylene and conventional polyethylene.

**Figure 2:**
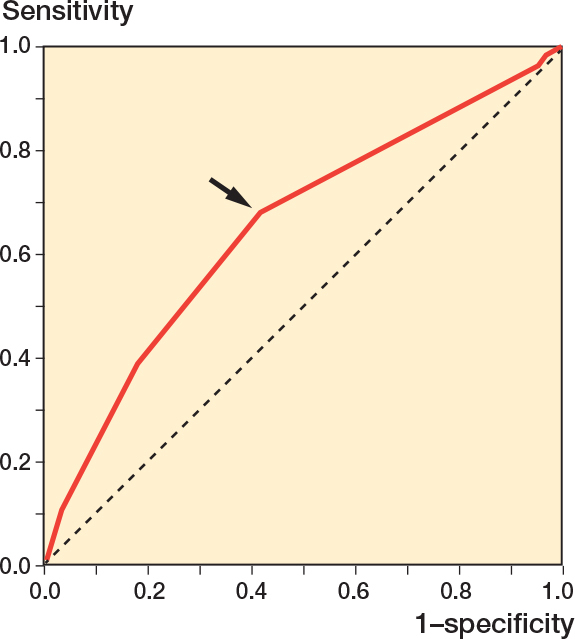
Receiver operating characteristic (ROC) curve plotting the true positive rate (sensitivity) against the false positive rate (1 – specificity) representing the portion of THAs with dislocation vs without regarding head diameter, which was treated as a continuous variable. On the curve, the point closest to the (0, 1) point is 30 mm in head diameter with sensitivity of 0.68 and 1 – specificity of 0.42 (arrow), which could be the threshold indicating ≥ 32-mm heads could reduce the dislocation risk. Area under the ROC curve is 0.65 (95% confidence interval 0.62–0.69).

The multivariable analysis identified younger patient age, higher BMI, posterior approach, and smaller prosthetic heads as risk factors (Table 3). As for patient age, the 1^st^ quartile (≤ 41 years) had a higher risk compared with the 2^nd^ quartile (42–51 years). There was no trend among age-divided quartiles (P = 0.09). The larger the BMI, the higher the risk of dislocation, but an appropriate threshold could not be found despite efforts to do so. There was a trend among the 3 approaches (P < 0.0001), i.e., in descending order, dislocation risk, posterior, lateral, and anterior or anterolateral approaches. Posterior approaches were associated with higher risks of dislocation compared with anterior or anterolateral approaches and with lateral approaches. There was a trend among head diameter groups (P < 0.0001). The bigger the head, the less likely to dislocate. However, ≥ 36-mm heads were not different from 32-mm heads. The results were robust to a sensitivity analysis applied to 2,854 hips performed for stage 4 (OA) ONFH (Table 4). However, in a sensitivity analysis applied to 3,129 hips performed for stage 3 or 2 (before the development of OA) ONFH, younger patient age was not a risk factor (Table 5). To decrease the influence of late dislocation, a sensitivity analysis was applied to 4,509 THAs with < 10-year follow-up (median 5.3 years), resulting in similar results except for younger patient age and previous hip surgery (Table 6). When sensitivity analysis was performed with the study group of 5,983 THAs, dividing the ≥ 36-mm group into 36-mm and 38-58-mm subgroups, neither of them was different from the 32-mm group (Table 7). On the ROC curve plotted to determine the threshold of head diameter that minimized dislocation risk, the point closest to the (0, 1) point was 30 mm in head diameter (Figure 2).

**Table 3:** Multivariable analysis applying the multivariable logistic regression model to the 5,983 THAs performed for ONFH

Variable	Odds ratio (CI)	P value
Patient age, years (ref.: 2nd quartile [42–51])		
1st quartile (≤ 41)	1.45 (1.02–2.07)	0.04
3rd quartile (52–62)	1.03 (0.71–1.49)	0.9
4th quartile (≥ 63)	1.08 (0.74–1.57)	0.7
Body mass index (ref.: increment of 1)	1.05 (1.02–1.08)	0.003
Previous hip surgery (ref.: no)	1.36 (0.93–1.98)	0.1
Surgical approach, posterior		
(ref.: anterior or anterolateral)	3.33 (1.96–5.56)	< 0.0001
(ref.: lateral)	2.27 (1.59–3.23)	< 0.0001
Incision length (ref.: conventional)		
Minimum incision surgery	0.90 (0.62–1.28)	0.5
Head diameter, mm (ref.: 32)		
≥ 36	1.06 (0.69–1.63)	0.8
28	2.30 (1.62–3.27)	< 0.0001
26	3.14 (2.18–4.53)	< 0.0001
22	6.81 (4.18–11.1)	< 0.0001

For abbreviations, see Table 2.

**Table 4:** Sensitivity analysis applying the multivariable logistic regression model to 2,854 THAs performed for stage 4 ONFH

Variable	Odds ratio (CI)	P value
Patient age, years (ref.: 2nd quartile [42–51])		
1st quartile (≤ 41)	1.88 (1.12–3.14)	0.02
3rd quartile (52–62)	1.06 (0.62–1.80)	0.8
4th quartile (≥ 63)	0.85 (0.48–1.51)	0.8
Body mass index (ref.: increment of 1)	1.05 (1.00–1.09)	0.02
Previous hip surgery (ref.: no)	1.34 (0.83–2.17)	0.2
Surgical approach, posterior		
(ref.: anterior or anterolateral)	4.17 (1.61–11.1)	0.003
(ref.: lateral)	1.85 (1.14–3.03)	0.01
Incision length (ref.: conventional)		
Minimum incision surgery	0.88 (0.51–1.50)	0.6
Head diameter, mm (ref.: 32)		
≥ 36	0.60 (0.28–1.30)	0.2
28	1.98 (1.17–3.34)	0.01
26	2.82 (1.66–4.78)	0.0001
22	5.71 (2.91–11.2)	< 0.0001

For abbreviations, see Table 2.

**Table 5:** Sensitivity analysis applying the multivariable logistic regression model to 3,129 THAs performed for stage 3 or 2 ONFH

Variable	Odds ratio (CI)	P value
Patient age, years (ref.: 2nd quartile [42–51])		
1st quartile (≤ 41)	1.01 (0.61–1.66)	1
3rd quartile (52–62)	0.99 (0.60–1.66)	1
4th quartile (≥ 63)	1.43 (0.87–2.35)	0.2
Body mass index (ref.: increment of 1)	1.05 (1.00–1.09)	0.04
Previous hip surgery (ref.: no)	1.25 (0.53–2.98)	0.6
Surgical approach, posterior		
(ref.: anterior or anterolateral)	3.13 (1.59–5.88)	0.001
(ref.: lateral)	3.03 (1.75–5.26)	< 0.0001
Incision length (ref.: conventional)		
Minimum incision surgery	1.00 (0.61–1.64)	1
Head diameter, mm (ref.: 32)		
≥ 36	0.60 (0.28–1.30)	0.9
28	2.81 (1.74–4.53)	< 0.0001
26	3.75 (2.22–6.34)	< 0.0001
22	9.95 (4.67–21.2)	< 0.0001

For abbreviations, see Table 2.

**Table 6:** Sensitivity analysis applying the multivariable logistic regression model to 4,509 THAs performed for ONFH and followed up for less than 10 years

Variable	Odds ratio (CI)	P value
Patient age, years (ref.: 2nd quartile [42–51])		
1st quartile (≤ 41)	1.44 (0.86–2.41)	0.2
3rd quartile (52–62)	1.18 (0.70–1.98)	0.5
4th quartile (≥ 63)	1.49 (0.91–2.44)	0.1
Body mass index (ref.: increment of 1)	1.04 (1.00–1.08)	0.03
Previous hip surgery (ref.: no)	1.84 (1.12–3.03)	0.02
Surgical approach, posterior		
(ref.: anterior or anterolateral)	2.63 (1.47–4.76)	0.001
(ref.: lateral)	1.96 (1.22–3.13)	0.005
Incision length (ref.: conventional)		
Minimum incision surgery	1.01 (0.65–1.58)	1
Head diameter, mm (ref.: 32)		
≥ 36	0.97 (0.60–1.55)	0.9
28	1.65 (1.06–2.56)	0.03
26	2.97 (1.81–4.88)	< 0.0001
22	4.65 (2.36–9.17)	< 0.0001

For abbreviations, see Table 2.

**Table 7:** Sensitivity analysis applying the multivariable logistic regression model to the study group of 5,983 THAs performed for ONFH dividing the ≤ 36-mm head diameter group into 36-mm and 38–58-mm subgroups

Variable	Odds ratio (CI)	P value
Patient age, years (ref.: 2nd quartile [42–51]		
1st quartile (≤ 41)	1.45 (1.02–2.07)	0.04
3rd quartile (52–62)	1.03 (0.71–1.48)	0.9
4th quartile (≥ 63)	1.08 (0.74–1.57)	0.7
Body mass index (ref.: increment of 1)	1.04 (1.00–1.08)	0.003
Previous hip surgery (ref.: no)	1.36 (0.93–1.99)	0.1
Surgical approach, posterior		
(ref.: anterior or anterolateral)	3.33 (1.96-5.56)	< 0.0001
(ref.: lateral)	2.22 (1.56–3.23)	< 0.0001
Incision length (ref.: conventional)		
Minimum incision surgery	0.90 (0.62–1.28)	0.5
Head diameter, mm (ref.: 32)		
38–58	1.19 (0.63–2.25)	0.6
36	1.00 (0.61–1.64)	1
28	2.30 (1.62–3.27)	< 0.0001
26	3.14 (2.18–4.53)	< 0.0001
22	6.82 (4.18–11.1)	< 0.0001

For abbreviations, see Table 2.

## Discussion

This is the largest prospective follow-up cohort study of primary THAs performed for ONFH. We aimed to evaluate risk factors associated with dislocation and whether larger head size could reduce the dislocation risk in patients with ONFH following a primary THA. In this nationwide study we show the following risk factors to be associated with postoperative dislocation: younger (≤ 41 years) patient age, higher BMI, posterior approach, and smaller prosthetic heads. Although larger heads were less likely to dislocate, 32-mm heads were large enough to reduce dislocation.

Compared with a report in 2004 [8], later reports indicated that first-time dislocations were concentrated in shorter periods after THA, probably due to use of larger heads: 81% within 12 weeks of surgery [12], 77% within 6 months [13], and 75% within 3 months [14]. Therefore, dislocation risk was analyzed in the 5,983 THAs with ≥ 0.5-year follow-up. Follow-up < 0.5 years was considered not long enough to assess dislocation.

### Patient age as a risk factor changed with time, and obesity

Patient age has changed as a risk factor. Previously, advanced patient age was reported as a risk factor associated with dislocation after THAs performed mainly for OA [5,8,9,14]. Recently, not older but younger age was reported as a risk factor [6,10]. In a survey in 2016 [7], with use of ≥ 32-mm heads in 34%, not only patients in the 4^th^ quartile (≥ 62 years) but also younger patients in the 1^st^ quartile (≤ 40 years) were at a higher risk of dislocation, compared with the 2^nd^ quartile. In the present study, with ≥ 32-mm heads in 57%, advanced age (the 4^th^ quartile, ≥ 63 years) was no longer a risk factor, but younger patient age (the 1^st^ quartile, ≤ 41 years) remained a risk factor (see Table 3). With increasing use of larger heads, transition from greater to younger patient age as a risk factor was observed in the former study, and in the present study, only younger age was a risk factor. Therefore, larger heads could have benefited older patients, thus lowering the dislocation risk. However, younger ONFH patients could still be at higher risk even with larger heads, possibly due to their increased activities with more frequent engagement of large and high-risk hip movements.

For the younger patient, age was a risk factor not in THAs performed for stage 2 or 3 (before development of OA) ONFH but for stage 4 (OA), indicating that this is not explained by decreasing hip motion with OA. It might be possible that cup position could be less accurate in the acetabulum with OA changes than without. In the sensitivity analysis applied to the THAs with < 10-year follow-up, younger patient age was not a risk factor but previous hip surgery became a risk factor. Dislocation risk for younger patients could become clearer in the longer term, requiring continued caution and education against dislocation. Patients who had had previous surgery in the index hip joint could be at a higher risk of dislocation, probably due to pre-existing soft-tissue damage in the shorter term until enough recovery of muscle strength.

Obesity has been reported to be a risk factor associated with dislocation after THAs performed mainly for OA [9]. In the present study of THAs performed for ONFH, the larger the BMI, the higher the risk of dislocation.

### Recently mitigated dislocation risk with posterior approach

Dislocation risk with a posterior approach has been mitigated. Previously, a posterior approach was reported as a risk factor associated with dislocation after THAs performed mainly for OA [5,15]. Recently, the risk was reported to be mitigated to the levels of other approaches [16,17]. This could be explained by repair of the posterior soft tissue [15] (especially the capsule [18]) and use of larger heads whose effect was greatest with a posterior approach [5]. Although the soft tissue repair technique was widely employed [7] and ≥ 32-mm heads were used in 57% in the present study, a posterior approach was still an independent risk factor associated with dislocation after THAs performed for ONFH.

### 32-mm heads were large enough to reduce dislocation

We showed that larger heads lowered the risk of dislocation in the multivariable analyses. However, ≥ 36-mm heads were not different from 32-mm heads. The ROC curve determined the threshold that best differentiated THAs with dislocation from those without to be a head diameter of 30 mm. The area under the ROC curve of 0.65 that indicates low accuracy may reflect the multifactorial nature of dislocation. However, in terms of head diameter, 32-mm heads were large enough to reduce dislocation in the present cohort of ONFH patients. This is reported here for the first time, to our knowledge. In biomechanical [19] and mathematical [20,21] studies, range of motion before impingement and jumping distance required for dislocation increased with increasing head size up to 28–32 mm, where the impingement shifted from component-to-component to bone-to-bone, becoming independent of head size and these benefits of larger heads were diminished. This may explain the present clinical results.

Several studies reported a reduction in dislocation risk with ≥ 36-mm heads. Decrease in dislocation rate in NHS (National Health Service) THA patients in England between 2005 and 2009 was explained by increase in use of ≥ 36 mm heads from 5% in 2005 to 26% in 2009 in the NJR (National Joint Registry) [22]. In 1 study, ≥ 36-mm heads were compared with ≤ 28-mm heads, but 32-mm heads were not included [23]. In another, ≥ 36-mm heads were compared with ≤ 32-mm heads, which must include 22- to 32-mm heads [6]. To our knowledge, only 1 study, which was done in Denmark has reported a higher risk of dislocation with 32-mm heads than with 36-mm heads after primary THAs [14]. In revision THAs, 36- or 40-mm heads reduced dislocation rates compared with 32-mm heads at 2- to 7-year follow-up [24]. In the present study, primary THAs performed only in Japanese ONFH patients were analyzed, the results of which require further research for external validity.

### Strengths

Demographic, surgical, and follow-up status information could be obtained precisely because of the prospective nature of the study, and the incidence rate of dislocation (which could be difficult to detect accurately in national registries) reflects the true value.

### Limitations

Prevalences of some categorical variables might not be enough to assess their effects on the risk. Some patient-related data were lacking, e.g., American Society of Anesthesiologists scores. Some operative data were missing, e.g., surgical experience [12]. Although the soft tissue repair technique was widely employed with a posterior approach in the present study [7], data specifically on capsule repair could not be obtained. Component position [12] could not be evaluated, given the constraints involving the 31 institutions. THAs performed only for Japanese ONFH patients were analyzed. Another limitation was the wide range of observation and follow-up periods. To reduce the influence of this, a sensitivity analysis was performed applying a multivariable logistic regression model to the THAs with < 10-year follow-up (see Table 6). Hip arthroplasty practice changed over the observation period in surgical approach, component fixation, acetabular articulating material, and material and diameter of the femoral head. All of the variables were included in the present analyses. Larger heads were used in patients with larger physique, except between the 36-mm and the 38–58-mm groups. The 2 groups were indicated to have been divided by institutional strategy. This could be a confounder. Although the prevalence of cobalt-chrome as acetabular articulating material (i.e., metal-on-metal THA) was higher in the 38–58-mm group than in the others, acetabular articulating material was evaluated in the univariable analysis (see Table 2) to be ineligible for the next multivariable analysis.

### Conclusions

We showed that younger patients with stage 4 ONFH (OA) and patients with higher BMIs are at higher risk of dislocation. These patients could probably benefit from surgical techniques that avoid a posterior approach and utilize larger heads up to 32 mm to reduce the risk of dislocation.

## Notes

SK, NS, WF, KK, and TS designed the study. NS, WA, and TS acquired funding and administered the project. SK, NS, WA, and TS contributed to data collection. SK, WF, and KK analyzed the data. SK wrote the first draft of the manuscript. All authors reviewed the manuscript.

The authors express their sincere gratitude to the orthopaedic surgeons listed at the following institutions who participated in this study. Institutions (researchers [currently working at other hospitals]) in geographical order from north to south: Asahikawa Medical University (Hiroshi Ito, Hiromasa Tanino), Hokkaido University (Daisuke Takahashi, Tomohiro Shimizu, Takuji Miyazaki, Takuya Ogawa), Sapporo Medical University (Satoshi Nagoya, Ima Kosukegawa), Yamagata University (Michiaki Takagi, Juji Ito), Chiba University (Junichi Nakamura, Shigeo Hagiwara, Yuya Kawarai), Dokkyo Medical University Saitama Medical Center (Tetsuya Jinno, Gaku Koyano, Ryota Shinada, Tetsuya Tachibana, Moe Suzuki), the University of Tokyo (Sakae Tanaka, Takeyuki Tanaka, Naoto Kaminaga), Tokyo Medical and Dental University (Ryohei Takada, Kazumasa Miyatake), Tokyo Medical University (Kengo Yamamoto, Takaaki Shishido, Toshinori Masaoka, Toshiyuki Tateiwa, Tsunehito Ishida), Yokohama City University (Yutaka Inaba, Hyonmin Choe, Hiroyuki Ike), Showa University Fujigaoka Hospital (Minoru Watanabe, Tsubasa Ishikawa, Itaru Kachi), Shinshu University ([Hiroyuki Kodaira], Hiroshi Horiuchi), Kanazawa University (Tamon Kabata), Kanazawa Medical University (Ayumi Kaneuji, Toru Ichiseki), Nagoya University (Yasuhiko Takegami, Yusuke Osawa), Mie University (Akihiro Sudo, Masahiro Hasegawa, Yohei Naito), Kyoto University (Yutaka Kuroda, Toshiyuki Kawai, Yaichiro Okuzu, Yugo Morita, Shuichi Matsuda), Osaka University (Kazuma Takashima, Keisuke Uemura, [Masaki Takao], Hidetoshi Hamada), Osaka National Hospital (Hidenobu Miki, Makoto Kitada, Yoshichika Hashimoto, Makoto Iwasa), Kansai Rosai Hospital (Takeshi Ogawa, Tsuyoshi Koyama), Osaka Metropolitan University (Yoichi Ohta, Ryo Sugama), Hiroshima University (Takeshi Shoji, Hideki Shozen, Shinichi Ueki, Hiroki Kaneta), Yamaguchi University (Takashi Imagama, Tomoya Okazaki, Yuta Matsuki, Takehiro Kaneoka, Takehiro Kawakami, Kazuhiro Yamazaki), Ehime University (Naohiko Mashima, Masaki Takao), Kyushu University (Yasuharu Nakashima, Goro Motomura, Ryosuke Yamaguchi, Takeshi Utsunomiya, Noriko Yamamoto, Hidenao Tanaka, Yusuke Ayabe, Kosei Sakamoto), Fukuoka University (Fumihiro Yoshimura, Yoshiaki Hideshima, Taiki Matsunaga, Hajime Seo, Koichi Kinoshita, Takuaki Yamamoto), Saga University (Masaaki Mawatari, Shunsuke Kawano, Masanori Fujii, Masaya Ueno), Nagasaki University (Makoto Osaki, Ko Chiba, Kyosuke Kobayashi, Kazuteru Shiraishi), Oita University (Nobuhiro Kaku), Miyazaki University (Etsuo Chosa, Takero Sakamoto, Yoichiro Yamaguchi), and University of the Ryukyus (Satoshi Nakasone, Masamichi Onaga, Takahiro Igei, Fumiyuki Washizaki).

Handling co-editors: Keijo Mäkelä and Robin Christensen

*Acta* thanks Georgios Tsikandylakis for help with peer review of this manuscript.

## References

[1] Thoen P S, Lygre S H L, Nordsletten L, Furnes O, Stigum H, Hallan G, et al. Risk factors for revision surgery due to dislocation within 1 year after 111,711 primary total hip arthroplasties from 2005 to 2019: a study from the Norwegian Arthroplasty Register. Acta Orthop 2022, 93: 593-601. doi: 10.2340/17453674.2022.3474. 35770369 PMC9244827

[2] AJRR 2024 annual report. Available from: https://connect.registryapps.net/2024-ajrr-annual-report (last accessed March 25, 2025).

[3] Australian Orthopaedic Association National Joint Replacement Registry. AOANJRR 2024 annual report. Available from: https://aoanjrr.sahmri.com/annual-reports-2024 (last accessed March 25, 2025).

[4] Tsikandylakis G, Kärrholm J, Hailer N P, Eskelinen A, Mäkelä K T, Hallan G, et al. No increase in survival for 36-mm versus 32-mm femoral heads in metal-on-polyethylene THA: a registry study. Clin Orthop Relat Res 2018; 476(12): 2367-78. doi: 10.1097/CORR.0000000000000508. 30260863 PMC6259897

[5] Berry D J, von Knoch M, Schleck C D, Harmsen W S. Effect of femoral head diameter and operative approach on risk of dislocation after primary total hip arthroplasty. J Bone Joint Surg Am 2005; 87(11): 2456-63. doi: 10.2106/JBJS.D.02860. 16264121

[6] Wyles C C, Maradit-Kremers H, Larson D R, Lewallen D G, Taunton M J, Trousdale R T, et al. Creation of a total hip arthroplasty patient-specific dislocation risk calculator. J Bone Joint Surg Am 2022; 104(12): 1068-80. doi: 10.2106/JBJS.21.01171. 36149242 PMC9587736

[7] Kobayashi S, Kubo T, Iwamoto Y, Fukushima W, Sugano N. Nationwide multicenter follow-up cohort study of hip arthroplasties performed for osteonecrosis of the femoral head. Int Orthop 2018; 42(7): 1661-8. doi: 10.1007/s00264-018-3980-1. 29754187

[8] Berry D J, von Knoch M, Schleck C D, Harmsen W S. The cumulative long-term risk of dislocation after primary Charnley total hip arthroplasty. J Bone Joint Surg Am 2004; 86(1): 9-14. doi: 10.2106/00004623-200401000-00003. 14711939

[9] Kunutsor S K, Barrett M C, Beswick A D, Judge A, Blom A W, Wylde V, et al. Risk factors for dislocation after primary total hip replacement: a systematic review and meta-analysis of 125 studies involving approximately five million hip replacements. Lancet Rheumatol 2019; 1(2): e111-21. doi: 10.1016/S2665-9913(19)30045-1. 38229338

[10] Zhang Z, Chi J, Driskill E, Mont M, Jones L C, Cui Q. Effect of patient age on total hip arthroplasty outcomes in patients who have osteonecrosis of the femoral head compared to patients who have hip osteoarthritis. J Arthroplasty 2024; 39(6): 1535-44. doi: 10.1016/j.arth.2023.12.029. 38135166

[11] Sugano N, Atsumi T, Ohzono K, Kubo T, Hotokebuchi T, Takaoka K. The 2001 revised criteria for diagnosis, classification, and staging of idiopathic osteonecrosis of the femoral head. J Orthop Sci 2002; 7(5): 601-5. doi: 10.1007/s007760200108. 12355139

[12] Biedermann R, Tonin A, Krismer M, Rachbauer F, Eibl G, Stöckl B. Reducing the risk of dislocation after total hip arthroplasty: the effect of orientation of the acetabular component. J Bone Joint Surg Br 2005; 87(6): 762-9. doi: 10.1302/0301-620X.87B6.14745. 15911655

[13] Dion C-A, Schmidt-Braekling T, Falsetto A, Kreviazuk C, Beaulé P E, Grammatopoulos G. Does surgical approach influence the natural history of the unstable total hip arthroplasty? J Arthroplasty 2022; 37(4): 787-94. doi: 10.1016/j.arth.2021.12.012. 34923093

[14] Hermansen L L, Viberg B, Hansen L, Overgaard S. “True” cumulative incidence of and risk factors for hip dislocation within 2 years after primary total hip arthroplasty due to osteoarthritis: a nationwide population-based study from the Danish Hip Arthroplasty Register. J Bone Joint Surg Am 2021; 103(4): 295-302. doi: 10.2106/JBJS.19.01352. 33347013

[15] Masonis J L, Bourne R B. Surgical approach, abductor function, and total hip arthroplasty dislocation. Clin Orthop Relat Res 2002; 405: 46-53. doi: 10.1097/00003086-200212000-00006. 12461355

[16] Maratt J D, Gagnier J J, Butler P D, Hallstrom B R, Urquhart A G, Roberts K C. No difference in dislocation seen in anterior vs posterior approach total hip arthroplasty. J Arthroplasty 2016; 31(9 Suppl):127–30. doi: 10.1016/j.arth.2016.02.071. 27067754

[17] Aggarwal V K, Elbuluk A, Dundon J, Herrero C, Hernandez C, Vigdorchik J M, et al. Surgical approach significantly affects the complication rates associated with total hip arthroplasty. Bone Joint J 2019; 101(6): 646-51. doi: 10.1302/0301-620X.101B6.BJJ-2018-1474.R1. 31154834

[18] Sun X, Zhu X, Zeng Y, Zhang H, Zeng J, Feng W, et al. The effect of posterior capsule repair in total hip arthroplasty: a systematic review and meta-analysis. BMC Musculoskelet Disord 2020; 21: 263. doi: 10.1186/s12891-020-03244-y. 32316961 PMC7175585

[19] Bartz R L, Nobel P C, Kadakia N R, Tullos H S. The effect of femoral component head size on posterior dislocation of the artificial hip joint. J Bone Joint Surg Am 2000; 82(9): 1300-7. doi: 10.2106/00004623-200009000-00010. 11005521

[20] Cinotti G, Lucioli N, Malagoli A, Calderoli C, Cassese F. Do large femoral heads reduce the risks of impingement in total hip arthroplasty with optimal and non-optimal cup positioning? Int Orthop 2011; 35(3): 317–23. doi: 10.1007/s00264-010-0954-3. 20157813 PMC3047653

[21] Sariali E, Lazennec J Y, Khiami F, Catonné Y. Mathematical evaluation of jumping distance in total hip arthroplasty: influence of abduction angle, femoral head offset, and head diameter. Acta Orthop 2009; 80(3): 277-82. doi: 10.3109/17453670902988378. 19421906 PMC2823207

[22] Jameson S S, Lees D, James P, Serrano-Pedraza I, Partington P F, Muller S D, et al. Lower rates of dislocation with increased femoral head size after primary total hip replacement: a five-year analysis of NHS patients in England. J Bone Joint Surg Br 2011; 93(7): 876-80. doi: 10.1302/0301-620X.93B7.26657. 21705556

[23] Ertaş E S, Tokgözoğlu A M. Dislocation after total hip arthroplasty: does head size really matter? Hip Int 2021; 31(3): 320-7. doi: 10.1177/1120700019898404. 31912749

[24] Garbuz D S, Masri B A, Duncan C P, Greidanus N V, Bohm E R, Petrak M J, et al. The Frank Stinchfield Award: Dislocation in revision THA: do large heads (36 and 40 mm) result in reduced dislocation rates in a randomized clinical trial? Clin Orthop Relat Res 2012; 470(2): 351-6. doi: 10.1007/s11999-011-2146-x. 22038174 PMC3254758

